# Supplementary figures and images for: NFYA regulates two sequential genome-wide transcriptional activation events during oocyte to embryo transition
Source: bioRxiv. 2026 Apr 1:2026.03.30.715371. Preprint. [Version 1] doi: 10.64898/2026.03.30.715371 (PMC13060261; doi:10.64898/2026.03.30.715371)

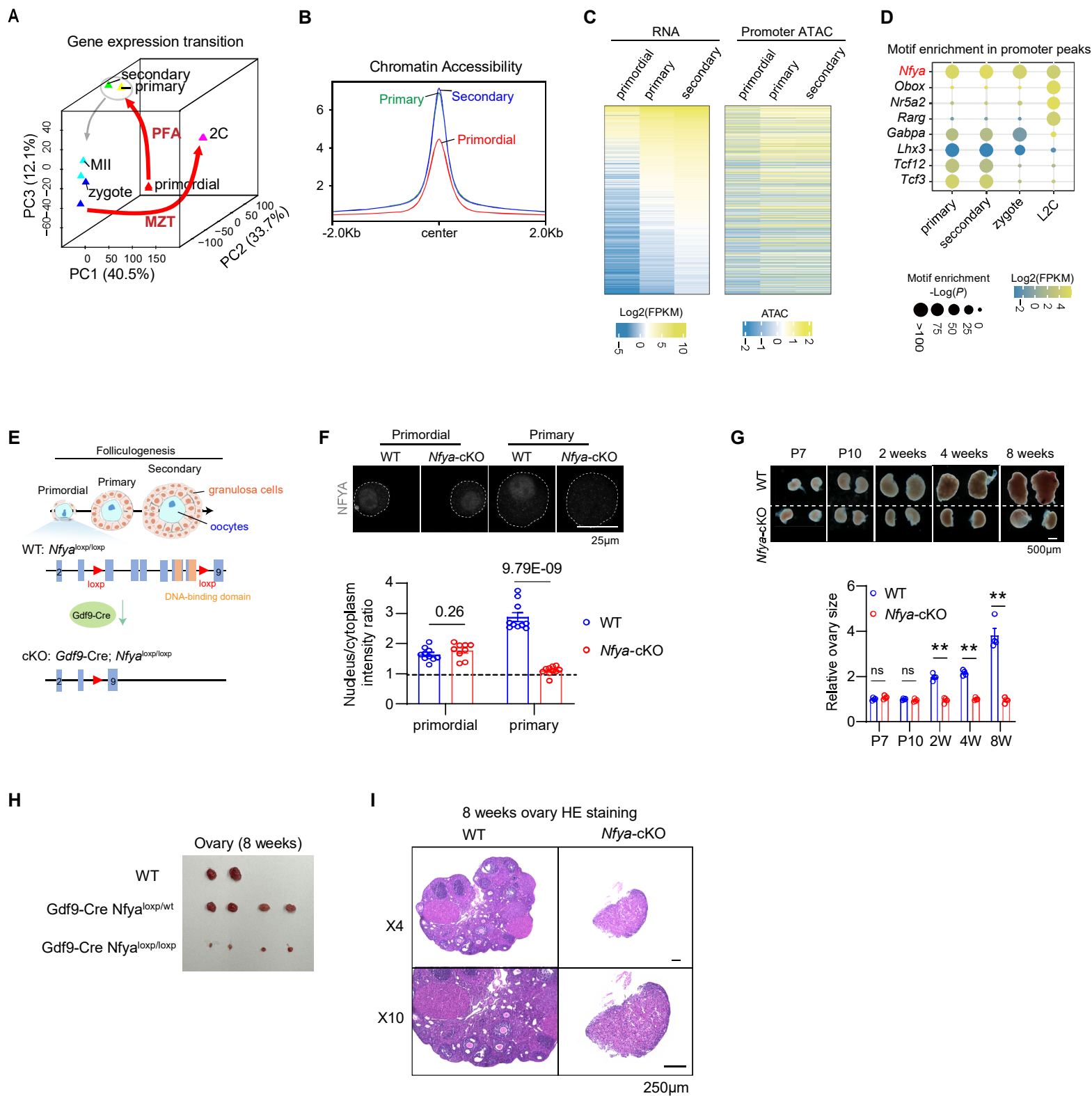

Fig. S1

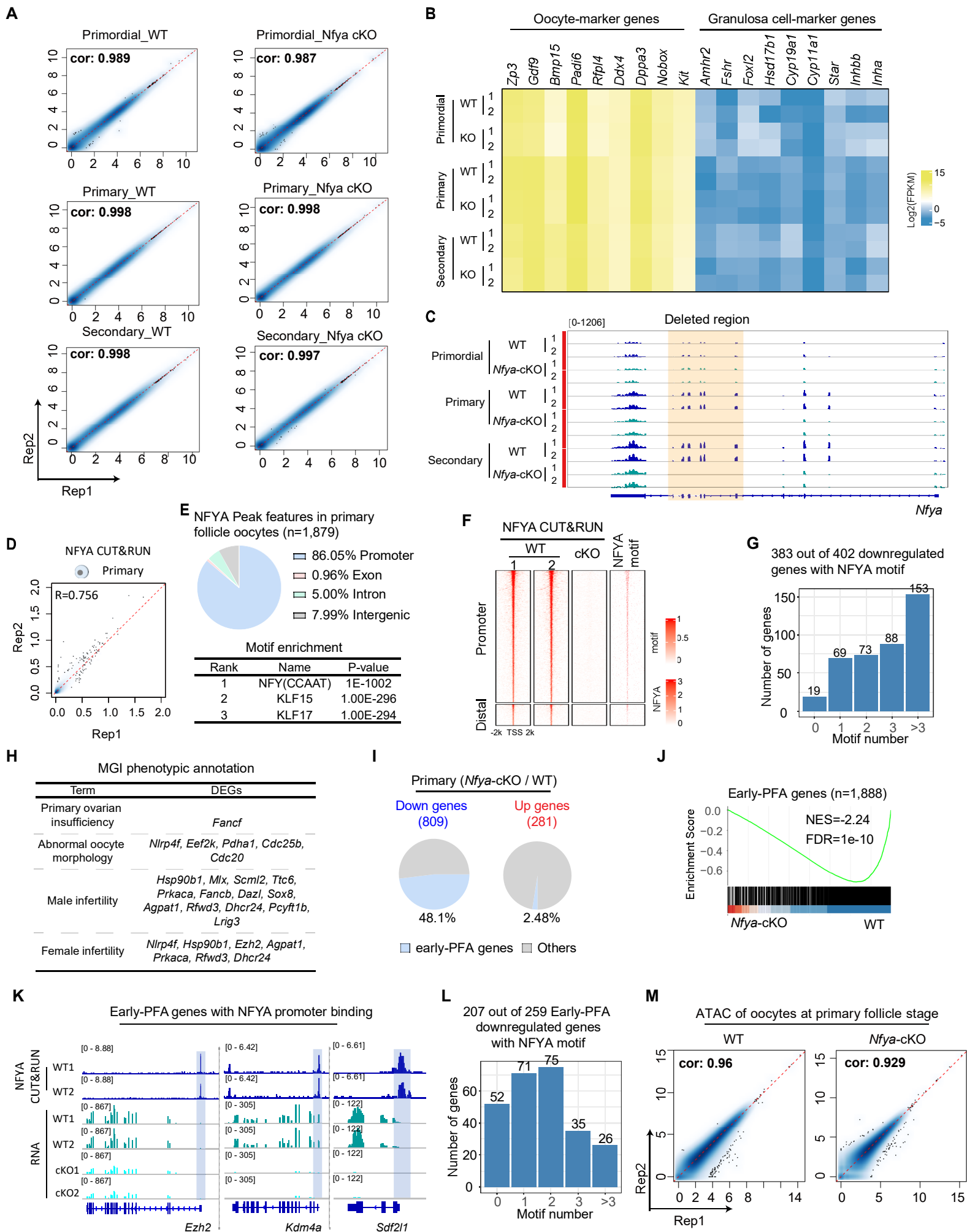

Fig. S2

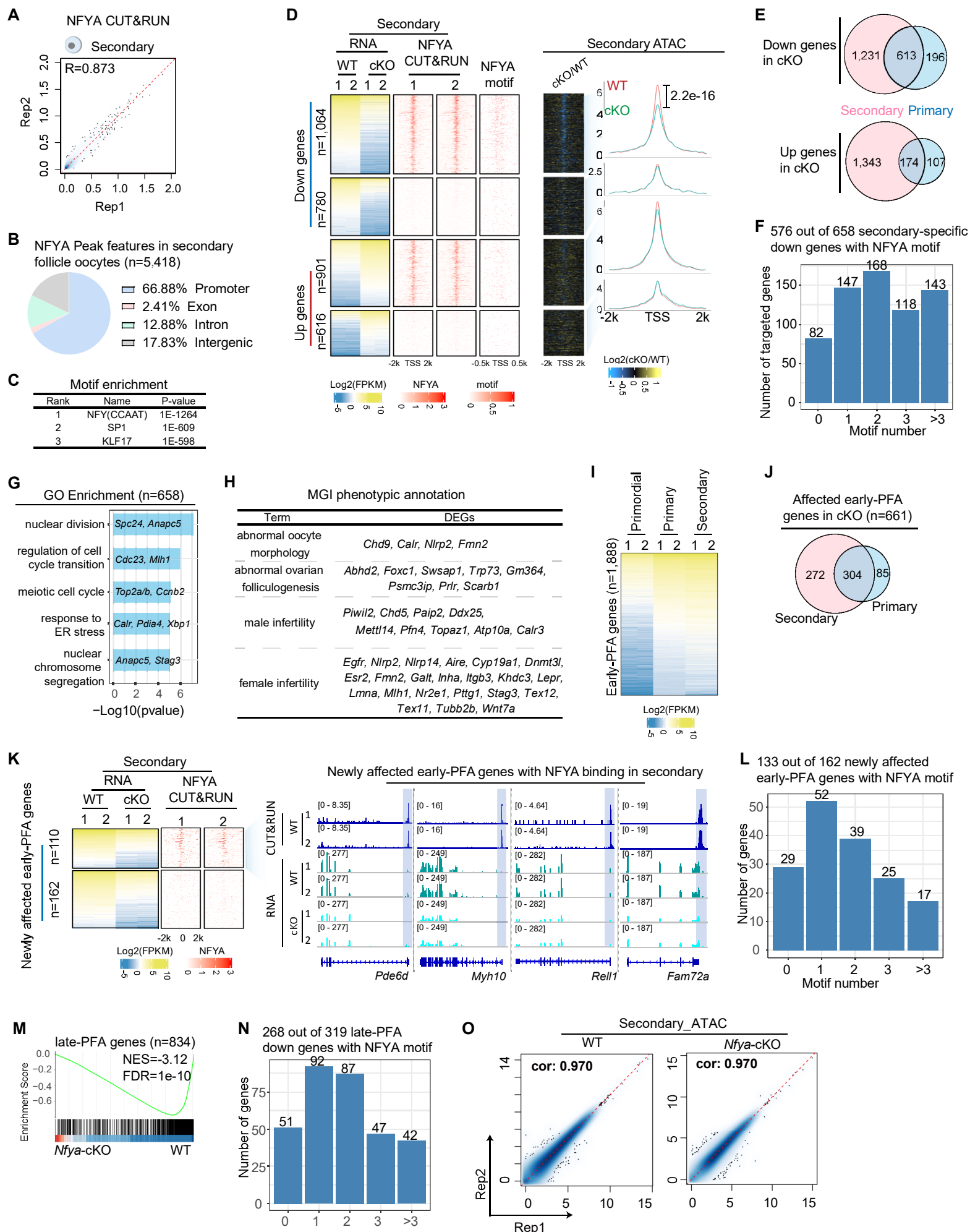

Fig. S3

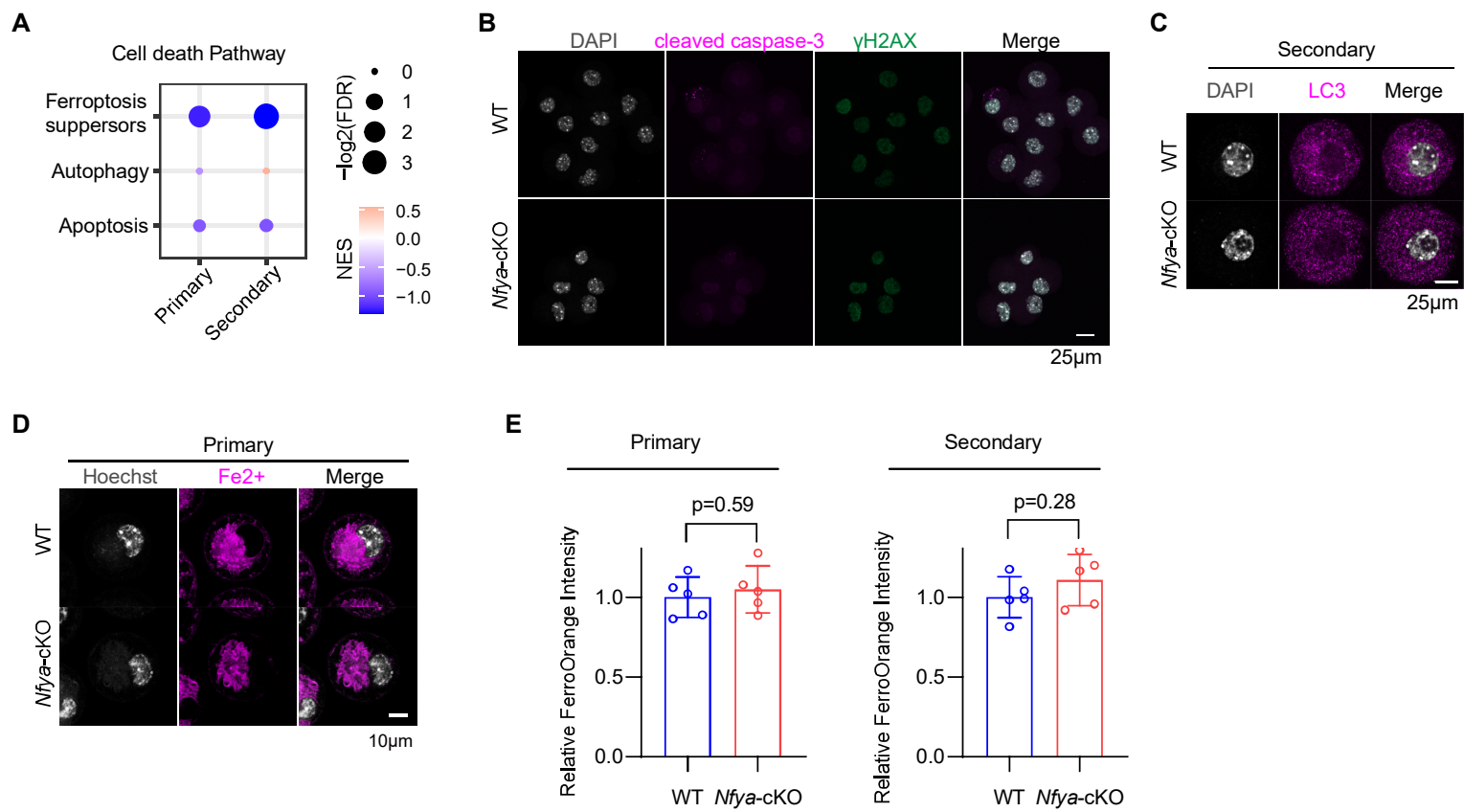

Fig. S4

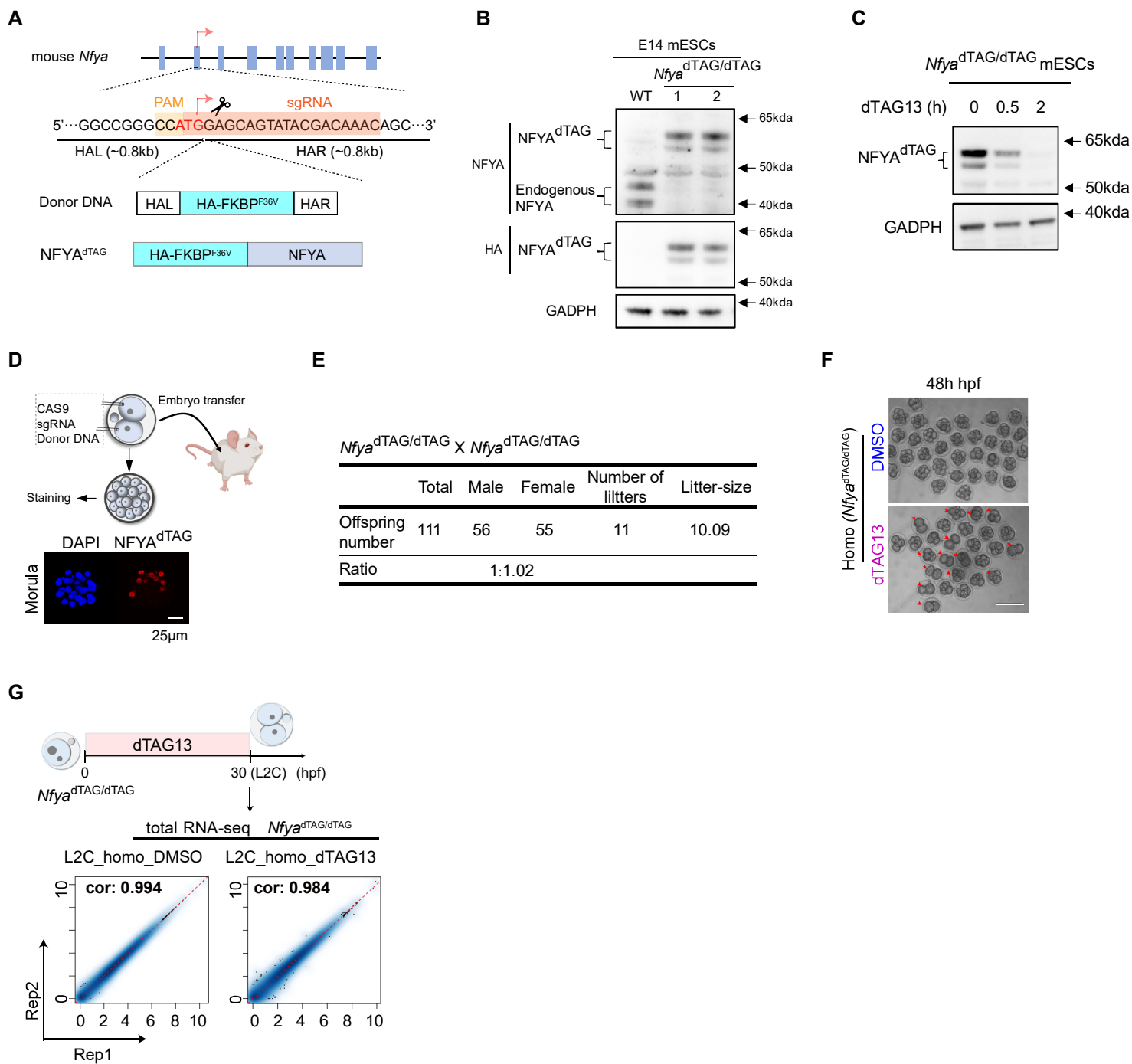

Fig. S5

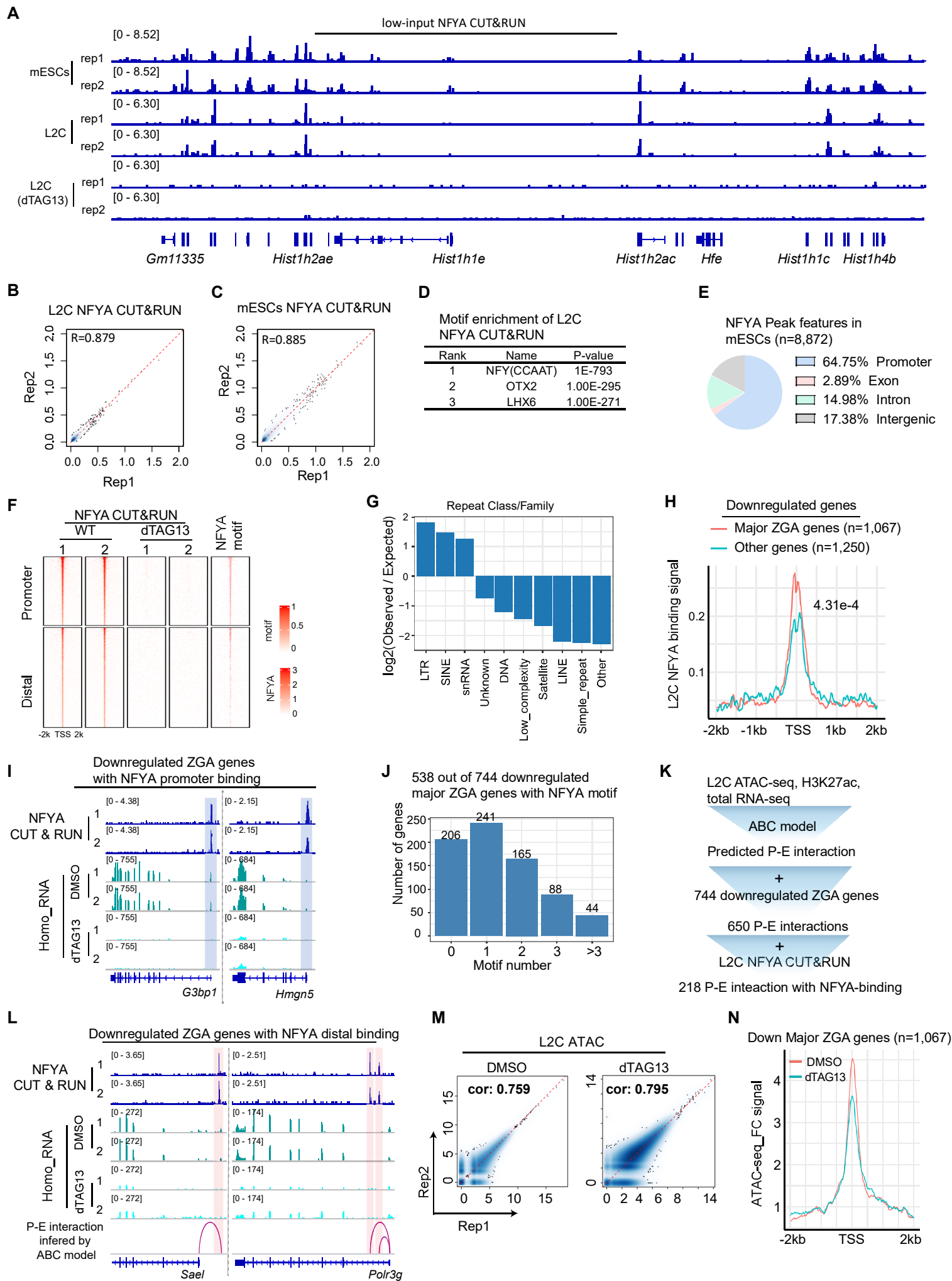

Fig. S6

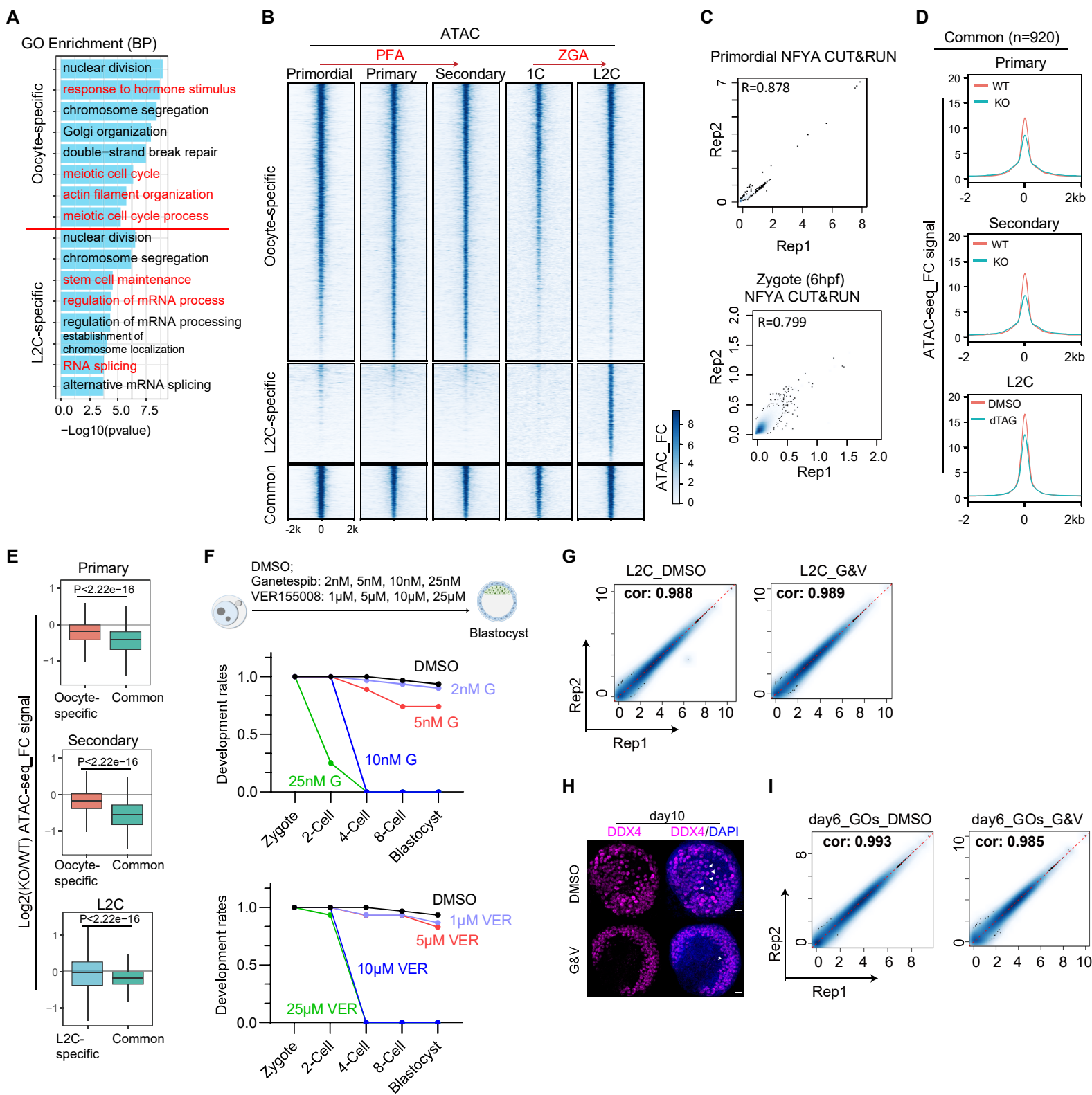

Fig. S7

Supplement: Supplement 1 — Figure S1. Identification of NFYA as a potential regulator of PFA and ZGA and demonstration that NFYA loss impairs folliculogenesis. (A) Principal components analysis (PCA) of gene expression from primordial follicle to late 2-cell embryos. (B) Metaplot showing the fold change of ATAC–seq signals (ATAC_FC) around transcription start sites (TSSs) of primordial, primary, and secondary follicles. (C) Heatmaps showing the expression of upregulated genes from primordial to primary and secondary follicles, and the corresponding ATAC_FC signals at their promoters. (D) Enrichment of TF motifs at promoter ATAC–seq peaks of the indicated stages. (E) Schematic diagrams showing the early folliculogenesis and a conditional Nfya knockout strategy using Gdf9-Cre-mediated deletion of exons 4-8 harboring its DNA-binding domain. Gdf9-Cre is specifically active in oocytes starting from primordial follicle stage. (F) Upper panel: the grey scale of NFYA signals in WT and Gdf9-Cre Nfyaloxp/loxp (Nfya-cKO) oocytes from figure 1G. Scale bar, 25 μm. Lower panel: relative nuclear to cytoplasmic intensity ratio of NFYA in primordial and primary follicle oocytes from WT and Nfya-cKO female mouse. P values, Student’s t test. (G) Upper panel: The gross morphology of ovaries derived from WT and Nfya-cKO mice at postnatal day 7 (P7), 10, 2 weeks, 4 weeks, and 8 weeks. Lower panel: the relative size of ovaries from WT (n=4) and Nfya-cKO mice (n=4). Scale bar, 500 μm. (H) Images of 8-week ovaries from WT (Nfyaloxp/loxp), Gdf9-Cre Nfyaloxp/wt, and Gdf9-Cre Nfyaloxp/loxp (Nfya-cKO) mice. (I) H&E staining of paraffin embedded 8-week ovarian sections of WT and Nfya-cKO mice. Scale bar, 250 μm. Figure S2. NFYA deficiency impairs PFA in primary follicle oocytes. (A) Correlations of the replicates of the total RNA-seq. The x and y axis of the dot plots are Log (CPM+1). (B) Heatmap showing the expression of oocyte- and granulosa cell-marker genes in the WT and Nfya-cKO primordial, primary, and secondary fol [file media-1.pdf]
