# Supplementary material for: NFYA regulates two sequential genome-wide transcriptional activation events during oocyte to embryo transition
Source: bioRxiv. 2026 Apr 1:2026.03.30.715371. Preprint. [Version 1] doi: 10.64898/2026.03.30.715371 (PMC13060261; doi:10.64898/2026.03.30.715371)
Supplement: 2 [file NIHPP2026.03.30.715371v1-supplement-2.pdf]

bioRxiv preprint doi: https://doi.org/10.64898/2026.03.30.715371; this version posted April 1, 2026. The copyright holder for this preprint (which was not certified by peer review) is the author/funder, who has granted bioRxiv a license to display the preprint in perpetuity. It is made available under aCC-BY 4.0 International license.

Supplemental tables (separated files)

Table S1: Differential expressed genes in primary follicle oocytes of *Nfya*-cKO versus WT

Table S2: Gene lists used in this study

Table S3: Differential expressed genes in secondary follicle oocytes of *Nfya*-cKO versus WT

Table S4: Differentially expressed genes of late two-cell embryos of NFYA$^{dTAG/dTAG}$ treated with dTAG13 versus DMSO

Table S5: Differentially expressed genes of late two-cell embryos of WT treated with G&V versus DMSO

Table S6: Differential expressed genes of growing oocytes from 6 days in vitro cultured P4 ovaries of WT treated with G&V versus DMSO

Table S7: Oligos used in this study

Table S8: ddPCR validated Nfya-dTAG knock-in copy number

Table S9: Summary of the sequenced libraries in this study

Table S10: Summary of the public datasets used in this study
